# Utilizing Genomically Targeted Molecular Data to Improve Patient-Specific Outcomes in Autism Spectrum Disorder

**DOI:** 10.3390/ijms23042167

**Published:** 2022-02-16

**Authors:** Sharon Hausman-Cohen, William LaValley, Heather Way, Emily Gutierrez, Jordan Reeder

**Affiliations:** 1 IntellxxDNA, Austin, TX 78731, USA; jreeder@intellxxdna.com; 2 LaValley MD Protocols, Austin, TX 78759, USA; jwl@lavalleymdprotocols.com; 3 The Australian Center for Genomic Analysis, Kenmore, QLD 4069, Australia; drheatherway@tacga.com.au; 4 Neuronutrition Associates, Austin, TX 78730, USA; egutierrez@neuronutritionassociates.com

**Keywords:** autism spectrum disorder (ASD), clinical decision support tool (CDS tool), SHANK3 (SH3 and multiple ankyrin repeat domains 3), genomics, molecular biology, variant, SNP (small nucleic polymorphism), personalized medicine, glutamate, PMS (Phelan-McDermid syndrome), PDD (pervasive developmental disorder)

## Abstract

Molecular biology combined with genomics can be a powerful tool for developing potential intervention strategies for improving outcomes in children with autism spectrum disorders (ASD). Monogenic etiologies rarely cause autism. Instead, ASD is more frequently due to many polygenic contributing factors interacting with each other, combined with the epigenetic effects of diet, lifestyle, and environment. One limitation of genomics has been identifying ways of responding to each identified gene variant to translate the information to something clinically useful. This paper will illustrate how understanding the function of a gene and the effects of a reported variant on a molecular level can be used to develop actionable and targeted potential interventions for a gene variant or combinations of variants. For illustrative purposes, this communication highlights a specific genomic variant, SHANK3. The steps involved in developing molecularly genomically targeted actionable interventions will be demonstrated. Cases will be shared to support the efficacy of this strategy and to show how clinicians utilized these targeted interventions to improve ASD-related symptoms significantly. The presented approach demonstrates the utility of genomics as a part of clinical decision-making.

## 1. Introduction—The Need to Shift the Paradigm to a Personalized Medicine Approach

Autism spectrum disorder (ASD) is a constellation of neurodevelopment syndromes that can present in various ways, including social, communication, or behavioral deficits [1]. 

The classic paradigm for medical treatments has generally been identifying a diagnosis without considering varying potential root causes, i.e., “the pill for an ill” approach. However, as science has progressed, it has become clear that underlying most diagnoses of chronic conditions, including ASD, individual differences provide an opportunity for more effective precision medicine. Precision medicine aims to identify and address the underlying molecular differences unique to everyone. Targeting identifiable, relevant gene pathways and networks specific to an individuals’ genomic and molecular profile is a strategy that has been shown to improve treatment responses [2]. This genomically targeted personalized medicine strategy is effective across many medical disciplines, including oncology and neurology [2,3,4]. Components that convey individuality are of paramount importance for the successful treatment of ASD. The challenge in medicine has been identifying these molecular differences, particularly in the case of mental health or brain-related disorders, since the blood-brain barrier (BBB) makes it challenging to measure proteins and other metabolites. With advances in research and the fields of genomics and molecular biology, many of these differences can now be identified. 

A significant challenge with genomic medicine lies in translating an individual’s genomics into an action plan, a patient-centered personalized medicine response. To accomplish this, the first step is to understand how the gene variants identified in an individual’s genomic profile work on a molecular systems level. Then, it must be determined if there is a way to intervene in a genomically targeted fashion. Understanding the relevant molecular biology is paramount for developing clinical intervention strategies to target the variants’ function, pathways, and gene–gene interactions. The various cofactors, regulators, and enzymes that degrade encoded proteins can be considered for therapeutic intervention. 

Addressing gene variants does not change the DNA itself but can affect the encoded downstream proteins via various mechanisms. How this can occur include the following: (1) increasing the transcription of the protein product by affecting transcription factors or increasing reaction speed; (2) making sure that co-factors that help push the reaction are plentiful; (3) adding factors that stabilize the protein products or decrease protein degradation; and (4) bypassing the affected gene product by identifying and promoting the synthesis of downstream reactions to gain the desired therapeutic effect. We will demonstrate how we used each of these strategies to develop potential interventions for individuals with a known SHANK3 variant to illustrate each of these strategies below.

## 2. Strategy for Identifying Modifiable Targets—Identify These Molecular Differences

Autism, like most chronic diseases, is a polygenic condition with different contributing variants found in other individuals. While over 2500 genetic mutations and variants have been associated with autism on some level [5], a relatively small number of variants have been identified and contribute to autism in a more significant way, increasing the odds ratio by 20% or more. The hypothesis in the building of the genomic clinical decision support (CDS) tool described in this paper was that identifying these higher-risk genomic variants could allow for a better understanding of contributing factors to an individual’s ASD. Once identified, strategies, as described below, could be utilized to modify and address these genomic factors. While this paper will share molecular insights used to develop interventions targeted at SHANK3, it is essential to understand that SHANK3 was not the only variant addressed in treating the individuals highlighted below. Thus, other gene variants will be discussed in the case studies listed. However, SHANK3 was the genomic target identified with the highest odds ratio for autism for these particular patients. Further details about the genomic CDS utilized in the cases below are discussed in the methods section. 

## 3. Identifying Targets for Therapeutic Intervention—Approach to Addressing Genomic Targets

The basic strategy undertaken in developing the genomic CDS was to investigate the molecular effects of the identified genetic contributing factors and the clinical translation of these effects. The same discovery methods were used to find ways to modulate the effects of the gene being evaluated as a contributing factor to ASD. The gene, SHANK3, will be used to demonstrate this method. Variants in SHANK3 have been well described in the literature as profoundly contributing to increased ASD risk [6].

Information on SHANK3 was curated from the peer-reviewed scientific literature to determine the function of both the gene and the effect the particular single nucleotide polymorphism (SNP) found in these cases creates. 

Shank3 is a scaffolding protein that is densely associated with postsynaptic glutamatergic synapses and is an important part of the postsynaptic density (PSD), a specialized protein-dense structure attached to the postsynaptic membrane. It acts as a master organizer of the PSD because of its ability to interact with multiple key synaptic components, including glutamate receptor complexes, anchoring proteins, and the actin cytoskeleton [7]. SHANK3 is a critical orchestrator of frontocortical function. Disrupted connectivity of the prefrontal areas may underpin the socio-communicative impairments observed in SHANK3 mutation carriers [8].

The variant described in this paper is the c.1304 + 48 C>T transition of the SHANK3 gene. This variant affects the methylation of a CpG island. Specifically, this variant alters a CpG dinucleotide to a TpG dinucleotide, which is thought to dysregulate SHANK3 expression. The area in which this alteration occurs has recently been shown to be an essential regulatory factor for a short SHANK3 isoform, the loss of which has been demonstrated to result in synaptic disorganization and ASD symptoms in mouse models [9]. CpG islands are often found in the promoter regions of genes and are essential transcriptional regulators. This variant has been shown to decrease transcription, decreasing SHANK3 protein levels and function [9]. Lower SHANK3 expression can cause the disorganization of synapses, dysfunction in synthesizing glutamatergic receptors, and impaired glutamate signaling. Glutamate is an essential signal for neuronal connectivity during development. SHANK3 is also necessary to properly produce dendritic spines and ion channels on post-synaptic neurons [6,9,10]. SHANK3 levels also influence the formation of synapses, synaptic maturation, and neuronal plasticity. Furthermore, SHANK3 interacts with other gene products to facilitate the long-term potentiation (LTP) and long-term depression (LTD) of hippocampal synapses [11]. This is a critical mechanism for forming neural circuits and, therefore, memory and learning [12]. Given the multitude of functions that depend on proper SHANK3 levels, it is not surprising that this variant in the SHANK3 gene has been associated with global developmental delays and significant issues with speech, as well as poor muscle tone [6]. It is highly associated with ASD, conveying an odds ratio (OR) of 5.4 for ASD [9] and a notably high OR of 12.3 for pervasive developmental disorder (PDD) [10]. 

After the SNP was identified, a literature search was conducted to help elucidate potential modification strategies. For many variants in the genomics tool, the gene mechanism and potential interventions were easily identified, as was the case for some of the specific inflammatory pathways, such as IL1B, that raised the production of IL1B. In the case of SHANK3, where the gene function is complex, a proprietary tool, the Moleculosity™ platform, developed by W.L., was used to efficiently identify and explore actionable targets of the molecular activity of the gene in question. This software tool efficiently interrogates PubMed/PubMedCentral and additional publicly available open-access bioinformatics datasets, including those focusing on genomics, transcriptomics, proteomics, and cell lines. The Moleculosity™ platform was then utilized to identify potential therapeutic natural products and re-purposed pharmaceuticals (prescription medications already available with other FDA-approved indications) for rational consideration in multiple potential genomically targeted clinical interventions. 

### 3.1. Intended Molecular Rationale for Interventions

For genomics to be clinically valuable, an understanding of the potential interventions supported by molecular rationale and science needs to be available to clinicians. The end goal for the CDS described in this paper was to present the genomic variants that have been identified in the literature as being associated with ASD and could be contributing to the patient’s phenotype, along with ways of addressing these genomic and molecular pathways. 

**Figure 1:**
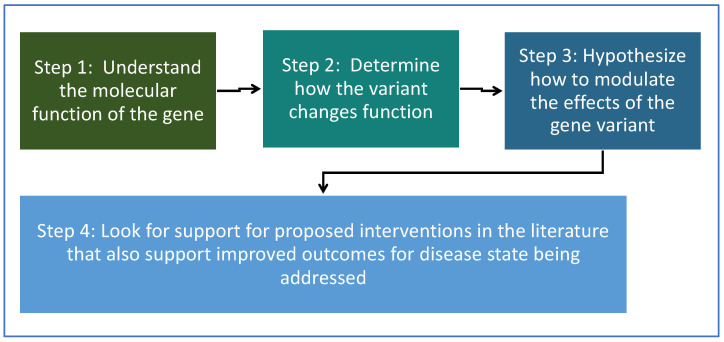
Overview of the approach used, from identification of a gene variant to presentation with potential interventions in the CDS.

For each gene variant, a basic approach illustrated in the flow chart below was taken (Figure 1). For step 3, in determining how to modulate the effects of the gene variant, four different potential approaches were explored. 

Possible ways to address genomic variants that were explored:

(1) Increase (or decrease) the protein concentration of the gene product.
(2) Address (increase or decrease) the regulatory factors and necessary co-factors.
(3) Protect the protein encoded by the gene from degradation by inhibiting degradation pathways.
(4) Address the downstream pathways and effects of the gene product to help mitigate or bypass the effects of the gene product.

#### 3.1.1. Increasing Protein Concentration of the Gene Product for SHANK3

Expression studies were evaluated to identify ways to increase the protein concentration of SHANK3. Studies show that SHANK3 expression levels vary throughout the day [13,14]. A study investigating the roles of physical activity and circadian rhythms in SHANK3 expression found that physical activity and light exposure were significantly associated with increased SHANK3 expression [13]. This mechanism appears to be, in part, that exercise increases melatonin. Melatonin was the circadian-modulating factor that increased SHANK3 levels [14,15]. While melatonin is classically thought of as a hormone triggered to be released during hours of darkness. Its regulation is related to complex circadian rhythms; both exercise and light exposure during the day positively modulate the release of melatonin at night. Light exposure also helps to trigger serotonin synthesis, and serotonin is the precursor to melatonin [16]. However, acute exposure to light, such as blue light before bedtime, can suppress melatonin release by mimicking daytime light and resetting the circadian rhythm.

In understanding this pathway, potential interventions were developed that supported SHANK3 expression related to circadian rhythms. This included supplementation with melatonin, using blue-light-filtering glasses during evening hours, avoiding screen time at night to lower blue light exposure, and utilizing blue-light-filtering devices on electronics used in the evenings. Avoiding blue light in the evenings helps maintain pineal gland function, circadian rhythms, and natural melatonin release [17]. Melatonin has been used in children to treat sleep disorders, which are commonly seen in cases of ASD. Melatonin has also been shown to improve some children’s behaviors with ASD [18]. Thus, because melatonin already had clinical evidence in ASD and an excellent safety profile, supplementation was deemed to be an appropriate potential intervention to list for this gene variant. Beyond supplementation, clinicians were also reminded to protect and promote endogenous circadian rhythms/melatonin synthesis with the blue-light-filtering interventions discussed above [13]. Studies have indicated a comorbidity between abnormal melatonin levels and ASD, with lower levels at a higher frequency in the ASD groups than in control subjects. This also supports that melatonin may play a role in the development of the symptomatology of ASD [18].

#### 3.1.2. Address Regulatory Factors

When addressing genomic pathways, the regulatory co-factors necessary for the gene product production must be considered. The availability of co-factors can be a rate-limiting step for the synthesis of protein products. Adequate or even high amounts of these co-factors can help to drive reactions. Zinc works on SHANK3 on many levels. Zinc acts to regulate and increase the expression of SHANK3, both by acting as a transcription factor and as a regulatory co-factor. Zinc deficiency has been shown to deplete synaptic pools of SHANK3 [19]. Thus, maintaining high zinc levels is crucial both during and after development for individuals with SHANK3 variants and is likely the most significant of all interventions identified to date for this pathway. Zinc is also the most well studied potential intervention for SHANK3. The literature provides clear evidence supporting zinc supplementation as a potential intervention for decreased function variants in this pathway [19]. 

**Figure 2:**
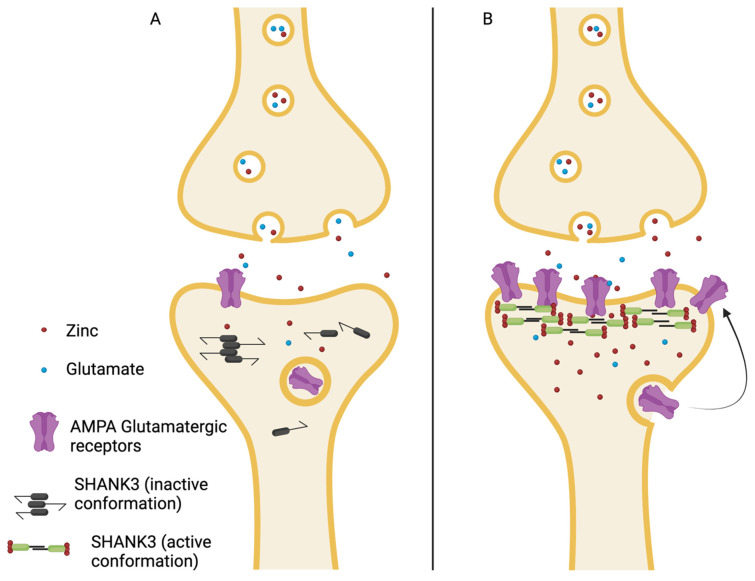
(**A**) In the presence of lower intracellular zinc levels, SHANK3 is inactive and can form oligomers (black SHANK3 on the left). With low zinc, there is lower recruitment and maturation of AMPA receptors. (**B**) Increased intracellular zinc levels allow SHANK3 to take an active conformation, forming a scaffolding protein and promoting the recruitment and maturation of synaptic AMPA glutamatergic receptors, thus increasing glutamatergic signaling pathways.

A simplified version of the mechanism by which zinc functions as a SHANK3 cofactor is that in a low-zinc environment, SHANK3 has an inactive conformation and often forms oligomers that impair its function as a scaffolding protein [20]. This leads to less glutamate AMPA synaptic maturation and impaired circuit formation [21]. In a high-zinc environment, the SHANK3- zinc complex can function as a scaffolding protein that allows for the “maturation” of synaptic AMPA-type glutamate receptors and, therefore, the proper development of glutamatergic signaling pathways. Zinc has been shown to increase the recruitment of synaptic AMPA (GluA2) receptors [18]. Figure 2 is a simplistic schematic that demonstrates this zinc-dependent conformational and functional change of SHANK3 leading to the proper development of glutamatergic signaling systems [20,22].

No genomically targeted prospective human studies utilizing zinc as a successful intervention for ASD in individuals with this SHANK3 variant have previously been published. However, the role of zinc as a potential contributing factor to ASD has been reviewed in many articles, and its importance in neurodevelopment is undeniable. A significant correlation (r = 0.367, *p* < 0.0001) between low zinc levels and ASD has been seen, particularly in young children [22,23]. The benefits of zinc in improving function in children with ASD have also been well-documented [24]. Additionally, there is strong animal model evidence for the use of zinc in SHANK3 depletion. 

In the mouse SHANK3 variant model, six weeks of dietary zinc supplementation ameliorated ASD-related repetitive and anxiety-type behaviors [19,22]. Furthermore, early evidence has been published for the use of zinc in Phelan-McDermid syndrome, which is a single SHANK3 deletion syndrome or haploinsufficiency, characterized by global developmental delays, decreased muscle tone, difficulties with or absence of speech, and ASD [20]. 

#### 3.1.3. Decrease Degradation of Protein Product to Increase Relative Protein Concentration

SHANK3 protein levels reach equilibrium and are determined based on a balance between the expression of the protein and the rate of degradation of the protein. Dysregulation of the degradation of SHANK3 can exacerbate the effects of SHANK3 dysfunction.

In search of the literature regarding regulators of degradation of SHANK3, a paper demonstrating that ERK2 is critical for the marking of SHANK3 for degradation was identified [25]. ERK2 phosphorylates SHANK3 to promote its ubiquitination, the “tagging” signal for degradation. The phosphorylation of SHANK3 causes the dissociation of SHANK3 from membranes, where it is degraded and, thus, cannot perform its scaffolding function. Inhibition of the ERK2 pathway increases SHANK3 abundance [25], and therefore inhibiting ERK2 has been proposed in the literature as a strategy to help to increase SHANK3 levels [25]. 

While prescription, highly targeted ERK2 inhibitors are available and currently used as therapeutics in treating melanoma and other cancers [26], these prescription medications have serious potential side effects [27] and are highly potent inhibitors. Given their side effect and safety profile and lack of studies in children, these would not be options that could be considered in children or individuals with ASD without extensive studies first demonstrating safety and efficacy. However, evaluation of the evidence-based literature showed several natural products to be mild inhibitors of ERK2. Understanding that ERK2 is a significant signaling cascade pathway, inhibiting it may have numerous effects. Thus, the list of potential ERK2 inhibitors was compared with the published medical and ASD literature to make sure that any supplements included as possible interventions had evidence for benefit in neurodevelopmental issues such as ASD in children, had good bioavailability, was able to cross the blood-brain barrier, and had an excellent demonstrated human safety profile in children. The ERK2 inhibitors with the best evidence for autism that met our criteria include quercetin, resveratrol, curcumin, and a butyrate-producing diet [28,29,30,31,32,33,34]. Each of these interventions has studies showing improved outcomes in children with ASD, and each of these has a GRAS (generally regarded as safe) rating and had previously been used in studies with children. These natural supplements were presented in the clinical decision support tool as potential interventions with references and detailed discussions.

#### 3.1.4. Address Downstream Pathways and Effects

As discussed above, SHANK3 impairs long-term synaptic potentiation, which is vital to neural networks necessary for learning and emotion. One strategy for addressing genomic variants with lower expression is to essentially “bypass” the issue by upregulating downstream affected pathways. 

Rather than working directly to increase levels of SHANK3 protein or affect stability, oxytocin is one such molecule that appears to work on downstream pathways such as long-term potentiation [35]. Increasing long-term potentiation (LTP) in multiple areas of the brain, including the hippocampus, has been shown to stimulate dendrite maturation and connectivity [36]. Decreased dendrite maturation is one of the possible downstream issues in individuals with SHANK3 deficiencies [9]. Due to its effects on LTP and its differential expression in the brain, oxytocin was also shown to decrease LTP-mediated pain hypersensitivity, often seen in individuals with ASD [36,37]. 

An animal study on SHANK3 deficient rats showed the benefits of intranasal oxytocin. Oxytocin is a hormone that has been associated with emotional regulation and social bonding. In some individuals with autism, lower plasma oxytocin has been noted [38]. Oxytocin has been shown to improve deficits in long-term social recognition memory attention and has been shown to increase synaptic plasticity in SHANK3 deficient animal models [35]. A small study of 18 children with SHANK3 haplotype insufficiency (Phelan-McDermid syndrome) has also been published using intranasal oxytocin with a *p*-value approaching, but not quite reaching, the threshold for significance at 12 weeks (*p* = 0.055). Changes on the Aberrant Behavior Checklist-Social Withdrawal (ABC-SW) was the endpoint measured in this study [39]. All of the above findings, along with the published studies of intranasal oxytocin being used and having a demonstrated benefit in children with ASD [40], made oxytocin qualify to be listed as a potential intervention for SHANK3 for the genomic CDS described. Additionally, microbial lysate from the probiotic lactobacillus reuteri has been shown to increase oxytocin levels in human studies, and, thus, it was also added to potential interventions [41]. One of the treating clinicians was in Australia, where oxytocin prescriptions are not readily available, so this nutritional probiotic bypass has been helpful for many of her patients. 

## 4. Proof of Concept Application of Tool: Case Studies

Ultimately, the goal of identifying genomically targeted interventions is to use a patient’s genomics to make personalized therapeutic suggestions. The patients then benefit by appreciating significant gains in function and improved outcome scores. This goal can only be accomplished by putting the identified interventions into a tool that the clinicians can easily access. In this case, the tool was a referenced and highly curated genomic CDS developed for this purpose. In the CDS, potential nutrients and other interventions identified as potential modification strategies were presented beneath the patient’s genotype and information about the gene variant so that clinicians could then choose amongst the targeted potential interventions and create a personalized, targeted plan for their patient. Specifics as to how this tool was used and how the SHANK3 and other targeted interventions benefited the patients can be seen in this series of SHANK3 case studies. Five out of the six individuals with SHANK3 made significant gains with this genomically targeted approach. For the sixth individual, other important environmental, dietary, and genomic factors were not addressed, which likely hampered progress.

### 4.1. Case Study 1

#### 4.1.1. Medical History and Background—Patient 1

This case study focuses on a thirteen-year-old male diagnosed with ASD at two. After receiving this diagnosis, the parents sought consultations and treatment options aggressively and had visited many specialized, well-respected autism centers and practitioners over the past 11 years. As part of his treatment over the years, many different treatment options had been tried, with some incremental improvements noted. The child presented to the clinician, E.G., with symptoms that have been summarized in Table 1, along with the treatment regimen at the time of presentation. 

After discussion and consenting to the risks and benefits, including that the CDS tool is not designed to diagnose or treat illness but is only for educational support of the clinician so that a more personalized clinical decision making can occur, the patient’s parents chose to pursue genomic testing. The IntellxxDNA™ neurodevelopmental report was ordered for this patient. In addition, blood labs, urine organic acids testing (OAT), stool studies, nutritional evaluation, and food sensitivity tests were also obtained. An action plan was formed in the following weeks to address the factors highlighted by these preliminary tests discussed above while the genomic results were pending. 

The patient was shown to have a high IgG response to dairy, gluten, and egg, and, thus, it was suggested that those foods be eliminated from his diet. Several nutritional deficiencies were identified and addressed, including him potentially being low on fiber, iron, vitamin B6, vitamin B12, methylfolate, and vitamin D. N-acetylcysteine was also added, due to test results suggesting low glutathione levels. Gut testing revealed that the child had markers of poor absorption and high lactoferrin levels, which can indicate intestinal inflammation [42]. In addition, this child was shown to have hyperlipidemia. To address these areas, in addition to the elimination of dairy, gluten, and eggs, micronutrients were repleted through supplementation and diet. Omega-3s were prescribed to address the cholesterol imbalances and for the general anti-inflammatory benefits. A mild improvement in symptoms was observed with these changes.

#### 4.1.2. CDS Genomic Results and Interpretation

Upon receiving the genomic results from IntellxxDNA™, multiple genomic variants were identified that could potentially contribute to the child’s ASD and neurodevelopmental symptoms. One of the first areas identified and addressed was the genomic variants contributing to various forms of inflammation. Inflammatory pathways overlap with many biological and regulatory processes in the body and have been associated with the symptomatology of many diseases and conditions, including ASD and ADHD [43,44]. Using the genomics and molecularly targeted natural products and therapeutic agents, the treatment of inflammation was personalized to the patient. For example, the patient had variants in the Interleukin 1-Beta (IL1B) gene associated with elements of brain inflammation and directly associated with ASD [45]. Both omega-3s and specialized pro-resolving mediators (SPMs, or resolvins) have been demonstrated to have a significant IL1B lowering effect. As discussed above, the child had already been started on omega-3s, but additional omega-3s were added when the child “flared.” Omega-3 supplementation is an effective strategy for improving behavioral characteristics in children with ASD and some aspects of ADHD [46,47]. 

This patient was also homozygous for another genomic variant that has been associated with elevated CCL2 (C-C motif chemokine ligand 2) levels [48]. Many natural products have been shown to lower CCL2 in the literature. Palmitoylethanolamide (PEA) was chosen for this patient, as a targeted intervention for decreasing CCL2. This particular intervention was chosen because of the published studies noting benefits in children with ASD [49]. The patient’s genomic results also reinforced what was seen in the labs regarding other specific nutritional deficiencies. The individual had significant SNPs known to contribute to low levels of Vitamin D, magnesium, and other micronutrients, some of which, such as magnesium, had already been addressed, since the clinician had conducted independent advanced nutrient testing. 

However, one of the most significant findings in this patient’s genomic results was one copy of the SHANK3 SNP, leading to the SHANK3 deficiency discussed above. Even one copy of this variant is associated with an almost six-fold increase in the risk of autism [9]. The CDS allowed the clinician to understand more about the role of SHANK3 as a crucial contributing factor to this child’s ASD. To address this pathway, a moderately high dose of zinc was chosen as one of the treatments to increase the protein concentration of SHANK3. The determined dose was 45 mg twice a day, similar to that studied in the pediatric literature, showing good safety and efficacy in children with ADHD [50]. In addition, the parents were instructed to limit the child’s exposure to blue light to promote proper melatonin production and to encourage physical activity. Prescription oxytocin was chosen as another treatment for this pathway. The patient also had variants in the oxytocin receptor gene (OXTR), which may have benefited from oxytocin replacement therapy. While SHANK3 was only one of many gene variants identified by the CDS that was addressed, it had the highest odds ratio of all the variants considered. Thus, it was deemed a highly significant piece of information allowing this clinician to develop a personalized treatment plan. The patient had several other SNPs that affected glutamatergic signaling, so the clinician also addressed these. 

#### 4.1.3. Effects of Implemented Interventions

Within a few weeks of introducing the genomically targeted interventions, this patient noted tremendous improvements. Excitingly, his mother indicated at the first follow-up visit that he was “able to go to school and enjoy it for the first time” and that he has been “calmer than ever.” This child’s functionality and quality of life have improved using molecularly targeted therapeutic agents based on this patient’s genomic profile. The parents reported that they were “thrilled with his progress” and that the genomically targeted interventions resulted in the “most gains in function that they had seen during his years of treatment.” Table 2, below, depicts the treatment plan following the genomics results and some outcomes noted by the parents and E.G. after six weeks of treatment using genomics.

The child has since followed up with the clinician, and his symptoms have noted further improvements. The parents specified marked progress in the year since beginning these targeted treatments. At follow-up, all of his abnormal labs had normalized. This included a significant reduction in inflammatory markers. Additionally, his growth and development are on track for a male his age. Even more exciting to the child and the parents are significant gains in social reciprocity. The child is now partaking in conversations that he previously would not have joined. At this point, the child’s most significant residual complaint is anxiety. Various treatment options, including SSRIs and other genomically targeted interventions, are being utilized. 

The child presented in this case is still undergoing treatment. His progress is being monitored and therapeutics are being shifted based on genomics, symptoms, and response, but his parents are highly pleased and excited with the progress. A limitation to this case study is that parental history and clinician exams were used to measure improvements, rather than formal quantifying scales. However, this cannot diminish the remarkable enhancement in this family’s life.

## 5. TACGA Case Studies

Under the guidance of Dr. Heather Way, the Australian Centre for Genomic Analysis (TACGA) has identified and treated several individuals with SHANK3 in a genomically targeted fashion. A closer look at these SHANK3 cases can help to elucidate some of the tremendous gains achieved when molecular insight is combined with genomic knowledge. The last case study also illustrates some of the limitations of this approach. 

### 5.1. Case Study 2

#### Previously Published Case—Patient 2

The first SHANK3 case identified by TACGA was previously discussed by Way et al. [2]. Patient 2 was a young man who had already been treated for his ASD, optimized, and had plateaued. He had been diagnosed with ASD at the age of 3. His IQ was measured at 54, giving him an additional diagnosis of intellectual impairment (II). This patient then presented to the Australian Center for Genomic Analysis (TACGA) at the age of 12. An initial ATEC (Autism Treatment Evaluation Checklist) was conducted, and he scored a 117. For reference, the average ATEC score for a 12-year-old boy is 28 [51]. TACGA worked with him over the years and reduced his ATEC score down to 71. It was still at 71 when they obtained access to the detailed neurodevelopmental genomic CDS. 

When his genomics came back, SHANK3 was addressed, as well as other contributing genomic variants, using high-dose zinc, melatonin, L. reuteri, and blue-light filtering options. The patient was also given mitochondrial supplements for additional identified variants. Tremendous improvements were noted for this child following these targeted treatments. His IQ increased by 16 points, and he was no longer classified as intellectually impaired. He became verbal, stopped his previously regular habit of nighttime bedwetting, was elected “class captain” in his high school, obtained his driver’s license and a paying job. His physical tone also improved, and he was able to start playing tennis and go to the gym. Equally impressive, his ATEC score, upon repeat testing a year after the genomic CDS was addressed, had normalized down to 21. He has now purchased his own car, regularly engages in meaningful conversations with others, has taken some classes at the local community college, and manages an online store for his mother’s business. He is now able to be an independent, productive member of society. 

### 5.2. Case Study 3

#### 5.2.1. Medical History and Background—Patient 3

The following case is of a 10-year-old boy with a regressive form of ASD, where milestones and speech had been lost. At the time of presentation, he exhibited significant developmental and language delays, problems with socialization, no eye contact, and anxiety and panic to the level where he could not sleep alone. He was also obsessed with electronic games. His speech was characterized by talking in the third person and problems with receptive language. He had difficulties explaining what he wanted, generally communicating with pointing. He would have flares of PANDAS (pediatric autoimmune neuropsychiatric disorders associated with streptococcal infection) where OCD-like behaviors, including tics, aggression, biting, and evidence of paranoid thoughts would be exhibited. Many of these symptoms were resolved when antibiotics for strep were given. He had been treated with five courses of antibiotics in the six months before presentation to the clinician. He had also presented on a gluten-free, dairy-free diet and had been on that for six years. The only ASD “specific” treatments he had been given were some “herbal tinctures” for immune support. Before addressing genomics, as per their usual protocol, TACGA worked on his gut microbiome inflammation and addressed his diet and nutrient deficiencies. Similar to the experiences of other clinicians, some improvements were seen with these interventions.

#### 5.2.2. CDS Genomic Results and the Effects of the Implemented Interventions

Genomic testing with the neurodevelopmental report was ordered. The results showed one copy of the SHANK3 variant. Other genomic variants contributing to difficulties synthesizing vitamin D on his own, problems with synthesizing glutathione, impaired melatonin signaling, and higher cortisol levels were noted. By November 2021, two months after genomic results were received and genomically targeted interventions for SHANK3 and other variants had been introduced, the patient was markedly improved. The mother reported extreme satisfaction with her child’s progress, improvements, and happiness since the introduction of the genomically targeted interventions. The mother says he is “doing well, and his ASD is getting better.” The child is going to school without his assistant. His teacher is shocked that he is doing so well without his aide. Behaviorally, he no longer exhibits hyperactivity, and his concentration is better. He is much more conversational, present in the moment, and even thinks of participating in the school robotics program. As an extra benefit, he sleeps well in his own bed, and the family enjoys spending time together. 

### 5.3. Case Study 4

#### 5.3.1. Medical History and Background—Patient 4

The following case is a three-year-old male who developed regressive ASD over a few weeks. The parents implemented the Nemechek protocol, a published autism protocol, with a different clinician but noticed no change in symptoms. The child presented to TACGA in August 2021. At that time, the most noticeable symptom was that he had a significant speech delay. He was classified as non-verbal, only able to use a few words, and his articulation of these few words was poor. He also exhibited visual stimming, toe walking, and made no eye contact. He was quite emotional and dysregulated. The parents and others described him as “spaced out, and socially isolated.” He, like many ASD children, was a picky eater. A GI Map stool test revealed he had C. difficile and Candida overgrowth in his microbiome. As per the TACGA protocol, in September of 2021, inflammation and gut were addressed, and some improvement in symptoms was noted. 

#### 5.3.2. CDS Genomic Results and the Effects of the Implemented Interventions

In early November 2021, when the genomics results were received, specific interventions for his heterozygous SHANK3 variant were addressed. This consisted of zinc, oxytocin, L. reuteri-containing probiotic, resveratrol, reducing screentime, and using blue light filters on devices to promote melatonin, as well as melatonin supplementation. The parents also turn off the Wi-Fi at night. A few other targeted interventions were added to support GABA pathways, B12 transport, and other pathways.

Within six weeks, the child started to talk. The mother reported significant improvements in his ability to emotionally regulate and progress in his socialization skills. As a bonus, he is now willing to eat a wider variety of foods. He still has significant OCD symptoms and stimming, but the specific interventions for the genomics related to these characteristics have not been fully addressed. The parents are thrilled with his rapid progress. 

### 5.4. Case Study 5

#### 5.4.1. Medical History and Background—Patient 5

The case of patient 5 is medically complex. Patient 5 was born prematurely, with several health issues resulting from prematurity complications. The patient had hypoxia at birth, retinal problems, and complications from anesthesia. The child also has had global developmental delays. 

In November 2020, at the age of 5, the child presented to TACGA with difficulties with socialization with children other than the immediate family unit, problems concentrating, appearing to be in their world, moderate developmental and speech delays, sensory issues, and motor issues (flexibility problems).

#### 5.4.2. CDS Genomic Results and the Effects of the Implemented Interventions

Patient 5 was evaluated using the genomic CDS described above and started on several interventions to address genomic contributing factors. These genomic factors touched upon and included difficulties with B12 transport and low b12 levels, variants that contributed to increased TNFa-related inflammation, problems with glutathione transferases, the removal of toxicants, choline synthesis, and several others. In addition, a heterozygous SHANK3 variant was present. At this point, the child was receiving zinc supplementation, but that was the only SHANK3 -specific intervention. In June 2021, a flare associated with an infection caused a setback and led to increased stimming and to being treated with several rounds of antibiotics. One month later, SHANK3-targeted interventions were introduced, including a higher dose of zinc, L. reuteri, blue light blocking options, and increasing his dose of curcumin. 

After three months of targeting SHANK3 more aggressively, the mother reported that the child is “doing amazingly,” and is noted to engage in more frequent and fluent conversation, with concentration level is improving. The mother also pointed out that the child is playing more with other children and is happier overall.

### 5.5. Case Study 6

#### 5.5.1. Medical History and Background—Patient 6

The last case presented here demonstrates the necessity of treating the genomic factors contributing to health conditions and addressing the environment around the individual. This case is of an 18-year-old who presented to TACGA in April 2021 with regressive ASD diagnosed at the age of 2. He was classified as non-verbal and demonstrated significant social and emotional regulation difficulties. He was noted to be “hard to control” and “needed to be watched all the time.” While he has a SHANK3 variant, this child also has other important contributing factors. He has two copies of an HLA DQ2.5, found in only 1.3% of the population, along with two copies of a rare variant in TNF-alpha, found in only 2.6% of the population, which is associated with an increased risk of gluten intolerance [52,53]. This combination of genomics gives him extremely increased risk of gluten sensitivity and celiac disease. Having 2 copies of HLA DQ2.5 has been associated with 1:7 risk of celiac disease vs. 1:100 in controls, which correlates to over 14x increased risk [52]. Additionally, he is homozygous for the TNFa SNP which has been associated with a 6x increased risk of celiac disease, compared to individuals without any of these TNFa variants [53]. Gluten sensitivity has been shown to exacerbate ASD [54]. The group home that he lives in serves a diet that is gluten heavy. This patient’s group home was found to contain mold. Mold exposure has also been described to cause neurological symptoms in sensitive individuals and may exacerbate ASD-like symptoms [55]. The child genomically also has problems with the removal of toxicants across the blood-brain barrier, specifically a gene variant in the ABCB1 pathway. This variant has been shown to be important for the removal of aflatoxin from the brain and may be important for other mycotoxins [56]. 

#### 5.5.2. CDS Genomic Results and the Effects of the Implemented Interventions

Upon receiving the genomic results in October 2021, SHANK3-specific interventions were started. L. reuteri, nasal oxytocin, liposomal melatonin, and blue-light-filtering options were provided for this child. As of December 2021, this young man has only demonstrated mild improvements in his symptoms. His sleep had mildly improved, but no dramatic changes in behavior, language, or intellect have been noted. He appears happy, busy, and can do well with the iPad and tasks with an aide, but he is still exhibiting significant verbal stimming. The family is looking to move the young adult to a facility that does not have mold and can provide a gluten-free and dairy-free diet. Given that this child has a high genomic predisposition to gluten intolerance, it is suspected that dietary and environmental triggers contribute to his ongoing symptoms and his poor response to the targeted interventions. It must be considered that the age at which treatment begins may affect treatment efficacy. The SHANK3 pathway, in particular, is heavily involved in early development, so age may demonstrate a role in treatment as well.

## 6. Sample of Other Genomic Variants Identified and Addressed

Several other genomic variants were found in varying combinations in the patients described above. The gene’s function and the effect of the mutation are included for each variant noted. This list of genomic variants is not a comprehensive list of variants identified for any one of these children, as the described CDS tool tests over 600 gene variants. A full list of the variants and the potential modifications utilized in this proprietary tool is released to clinicians as part of their training on the tool. Thus, a sampling of some of the most important variants identified and addressed in these cases is listed in the table for illustrative purposes. Several of these genetic variants are described in Table 3. 

## 7. Discussion

While the science of identifying and addressing modifiable genetic contributing factors is still in its infancy, significant evidence has accumulated supporting a genomically targeted personalized medicine strategy. As illustrated by the case of SHANK3, understanding the molecular mechanisms of a gene can lead to the identification of genomically targeted interventions. This molecularly and genomically guided strategy can translate to meaningful improvements for individuals. While the first half of this strategy identifies genomic contributing factors, the second, but very important, part of the process is to use molecular biology and current scientific literature to develop potential modification strategies. The power of molecularly targeted therapeutic agents has been shown and continues to show promise for these individuals and their families. 

Precision medicine tools open the horizon to targeting an individual’s molecular vulnerabilities. This allows for the utilization of targeted interventions of natural products or repurposed pharmaceuticals as personalized interventions. This strategy is commonly used in cancer drug design. While this article focuses on the benefits of genomic medicine for ASD, the same rationale and approach can be applied across the board to chronic illnesses. The underlying principle of this approach is that interventions are targeted to underlying genomic and molecular pathways rather than a diagnosis. 

While one gene variant was focused on in this discussion, this method must be applied to a wide variety of an individual’s contributing genetic variants simultaneously to ensure maximal success. The cases presented here demonstrated this idea. The need for this multi-pronged approach relates to the etiology of ASD. Autism, like most chronic illnesses, is generally due to the interactions of multiple gene products, the individual’s environment, lifestyle, and the interrelationships between these factors, rather than being monogenic in cause. 

There are no targeted pharmaceutical interventions available for children with SHANK3 variants. As illustrated in the provided cases, utilizing readily available supplements that are generally regarded as safe (GRAS), including natural products, vitamins, lifestyle, and dietary changes, to target SHANK3 and other underlying identified genomic factors, can allow clinicians to help children currently suffering from symptoms. Meanwhile, more specific, molecularly targeted interventions can be explored and developed via traditional pharmaceutical pathways. 

One limitation to this study is that the molecular pathways that play a role in ASD are vast and intertwined. In the same sense, the therapeutic agents selected to intervene in these pathways will affect a wide array of systems throughout the body in addition to the targeted gene. It is, in fact, likely and possible that the interventions chosen to modulate the SHANK3 gene affected more than just the targeted gene. For example, zinc is a co-factor to many hundreds of reactions throughout the body [65]. In addition to stabilizing SHANK3, zinc supplementation will also affect many other protein pathways. Thus, the therapeutic benefit and full extent of the interventions cannot be entirely known. Molecular investigation sheds light on some aspects of how genes interact with these agents, but the sheer complexity of the human body requires more in-depth research and evaluation.

Another limitation to this study is the size of the database. Above, we present data from a handful of SHANK3 cases. While the authors have successfully applied this approach to several individuals with SHANK3 and many hundreds of individuals with other ASD-contributing genomic factors, it is still a relatively small dataset. 

Additionally, data supporting a genomically targeted approach is primarily from observational case studies, rather than prospective, controlled trials. An important next step would be to do more extensive and more formal prospective, controlled studies with individuals with SHANK3 and other variants. As the use of genomic sequencing and genomic clinical decision support tools becomes more prevalent in ASD, it will become easier to identify candidates for this sort of study in a university or similar setting. 

## 8. Conclusions

Autism is a chronic medical condition with no clear approved treatment options and multiple contributing etiologies. The association of genomic variants with autism has been described in the literature in numerous articles for over a decade. However, previously, there has not been a tool or method available for translating genomic information into something that is useful or actionable to the clinicians treating individuals with autism. In conclusion, the most important implication of this work is that by allowing a clinician to better understand their patient on a molecular level, outcomes in autism can be genomically and molecularly targeted and improved. This is conducted utilizing a research method that has been, and will continue to be, used for additional chronic diseases [66]. This approach has the capacity to transform the practice of medicine into a precision science.

## 9. Potential Implications for the Genomically Targeted Approach to Neurodevelopmental Disorders

A molecularly targeted genomic approach provides clinicians with a new paradigm for treating chronic diseases. This strategy offers the ability to look at root causes and, equally importantly, provides a framework so they can address these genomic factors that have been shown to contribute to chronic illness, using a personalized molecularly targeted approach. 

This strategy can transform the practice of medicine and the treatment of chronic disease. Case study evidence provides significant support for this approach for individuals with ASD and cognitive decline [2,3]. This personalized approach can be applied to any number of chronic diseases and empowers both the clinicians and the patients, helping the patient feel confident that the clinician’s conclusions are relevant. A genomically targeted approach has been made possible, due to advancements in our understanding of molecular science and improvements in technology that have made genetic testing more affordable and available. These technological and scientific advances have allowed for a tool, such as the CDS presented here, to be developed to manage and support clinical decisions. 

The potential implications of modifying even just the trajectory of children with SHANK3 variants are significant. The presented case studies and the information gathered from the parents of children with ASD offer further insight. Naturally, genomic evaluation has shown the presence of the same SHANK3 variant discussed in this paper in many parents of affected children. In these parents, there is no evidence of intellectual disability or neurological concerns, despite this variant being purported to be associated with intellectual disability [6]. Some of these SHANK3-carrying individuals are successful physicians, Ph.D.’s, and lawyers. This supports the idea that this variant and many genetic contributing factors to ASD are modifiable and not pathogenic.

A further history of high-functioning adults carrying this SHANK3 variant revealed that many had lifelong diets high in zinc-laden foods, such as oysters and organ meats. Genomics allows for the identification of individuals for preventative and therapeutic effects. For example, individuals with SHANK3 variants would benefit from a higher zinc intake starting at birth or even prenatally if a parent has a known variant. 

While the field of genomically targeted personalized medicine is still in its infancy, the implications for specifying recommendations for nutrients, supplements, lifestyle, and even medications down to the individual level have the potential to change how medicine is practiced and improve patient outcomes. 

As illustrated by the cases above, dramatic results can be seen using genomically targeted molecular medicine in some circumstances, and in others, less dramatic results are seen. Not all children are going to obtain an equal magnitude of improvement, with variables such as their diet and environment contributing and the combined constellation of genomics that the child possesses. ASD rates have skyrocketed over the past decade. A study published in 2020 showed that 23 out of 1000 children suffered from ASD [67]. Initial results, as reflected in the cases above and in communications from clinicians utilizing this approach, are highly optimistic, reporting significant gains in functional outcomes with a genomically targeted approach in many of their patients with ASD. However, even if only 20 or 30% of children with ASD made noticeable gains utilizing this approach, that would still provide tremendous advances in the medical care of children with ASD. A genomically targeted approach is highly cost-effective, as an individual’s genomics do not change and can be seen as a roadmap for supporting an individual for life. 

Under the current medical system, many children with ASD become dependent on society to support them and do not achieve complete financial or social independence. For this reason, this strategy may be a highly cost-effective method not only for the patient but also for society. As illustrated by some of the case studies, evidence demonstrates the potentially remarkable gains individuals can make utilizing this approach, leading to an increased ability to become a productive member of society rather than a burden. More formal exploration and study of this approach is warranted.

## 10. Materials and Methods

The genomic targets included in the CDS were identified by searching the published medical literature. The genomic targets were selected primarily through literature and database searches, including PubMed, MDPI, LitVar, and dbSNP. Information was gathered regarding SNP allelic frequencies. While many gene variants have been associated with autism, rare deleterious and pathogenic variants, such as those found in <1% of the population, were purposely not included. This exclusion of low-frequency and pathogenic variants was due to the idea that ASD can develop based on the combinatorial effects of multiple variants found in higher frequencies.

To create a manageable report in size and length, an arbitrary cut-off was set for our ASD research panel to only include variants that had been shown to increase the risk of ASD by 20% or more, correlating to an odds ratio of ≥1.2. Additionally, a second panel of gene variants was developed. This panel consisted of gene variants with excellent research supporting an association between these variants and ASD or other neurodevelopmental conditions. No ORs were calculated, but a high statistical power was demonstrated for these variants. Frequently, these are from observational analysis data from children with ASD. Variants were only included in the genomic CDS if, upon further exploration, the mechanism of the gene had been previously elucidated in the literature and if it was felt to be a potentially modifiable target.

In building the CDS, the gene function for each gene was researched, along with how the variant associated with ASD in the literature was thought to affect the gene function. Then, a deeper literature search was conducted, again using the various resources mentioned above, to gain a molecular understanding of these pathways. In the case of SHANK3, as with many SNPs, the functional consequences of the SNP on gene function were not easily identified. W.L. was consulted to identify these various targets and methods to modulate the pathway. Dr. LaValley is a physician and molecular biologist who utilizes evidence-based molecularly targeted natural product supplements and re-purposed pharmaceuticals for molecular integrative medicine treatment of patients with complex chronic diseases. The research team then integrated this information into the genomic CDS for accessibility by the clinicians.

This molecular understanding of these pathways was then applied to the development of genetically targeted potential interventions for these patients with ASD, as is described in detail concerning SHANK3. 

The individuals’ clinicians ordered the genomic testing after parents consented to the risks, benefits, and limitations. Buccal swab sample collection was performed by the clinician’s medical staff or by the parents of the children. The samples were then sent to RUDCR, a CLIA CAP certified lab, where the DNA was purified and processed. Sequencing was conducted using a targeted custom array developed on the Affymetrix precision medical backbone. For gene variants of interest that had not been validated as having a quality threshold of >95% accuracy, PCR was also conducted. The genomic results were then presented to the clinician in the format of a genomic clinical decision support tool (CDS) that provided gene function, clinical relevance, and evidence-based potential interventions. Further details on this genomic CDS (IntellxxDNA) have been described elsewhere [2].

## Figures and Tables

**Table 1:** Summary of the patient’s reported clinical symptoms and treatment regimen before ordering genomic CDS. The symptoms and therapeutic regimen are noted below.

Primary Symptoms Reported	Treatments, Prior to Genomic Testing
Aggression/Rage Delayed speech Difficulty with switching tasks Inflexibility Introverted Overweight Picky eater Poor social skills Difficulties with emotional engagement	CBD/THC 3:1 ratio, as needed Methylphenidate, 4 mg daily High olive oil diet Prebiotic inulin Sertraline, 150 mg daily After nutritional studies came back, the following were added Omega-3s B vitamins NAC Vitamin D

**Table 2:** Summary of the treatments prescribed after genomic testing and the improved outcomes after six weeks on these treatments. Bid = bi-daily; * = some improvement; ** = notable improvement.

Treatments, Post-Genetic Testing	Outcomes of Personalized Treatment, after 6 Weeks
N-Acetylcysteine Continue dairy-free gluten-free diet Melatonin, 1mg at night Vitamin D3 Increasing dietary fiber Omega-3s Methylfolate, vitamin B6, and vitamin B12 combination supplement Zinc, 45 mg bid Sulforaphane with myrosinase Liposomal glutathione Oxytocin Sertraline, 100mg twice daily Palmitoylethanolamide Specialized proresolving mediators (resolvins) Ibuprofen, as needed Amantadine	Grudge-holding resolved * Aggression/rage resolved much easier ** Lower obsessive-compulsive symptoms * Healthier gut ** Tolerant of new dietary changes ** Healthy weight loss ** Significant improvements in verbal communication *

**Table 3:** A sampling of genetic variants that were found in the patients described above. Superscripts next to the gene symbol represent the presence of the variant in the corresponding patient’s case (1 = Patient 1, 2 = Patient 2, etc.).

Gene and Variant	Gene Function	Variant Effect
TNFa ^2,5,6^; −308G>A	TNFa is a pro-inflammatory cytokine that helps regulate the cytokine cascade of inflammation.	This variant results in increased transcription and production of TNFa. This SNP is in the major histocompatibility complex and has been associated with autoimmune diseases such as celiac disease [52].
IL1B ^1,2,3,4,5,6^; −511C>T	IL1B is a pro-inflammatory cytokine involved in early development. Additionally, IL1B stimulates other pro-inflammatory cytokines, such as IL6, and can cross the blood-brain barrier, which can result in alterations of neuroendocrine function.	This variant results in increased IL1B, which can lead to neuroinflammation. This inflammation in the brain has been associated with decreased neurogenesis and increased stress, anxiety, and social interactions [57].
OXTR ^1,3,5,6^; 690C>T	OXTR is the receptor for the hormone, oxytocin. Oxytocin is involved in regulating social behavior and bonding, as well as playing a role in reproduction and childbirth.	This variant, located in the promoter region, is suggested to alter oxytocin signaling. Lower oxytocin signaling has been associated with decreased pair-bonding and social skill scores. This variant has also been associated with impaired social cognition, as can be seen in ASD [58].
TCN1 ^1,2,5^; D301Y	TCN1 is a vitamin B12 binding protein that aids in the protection of B12 from digestive acids in the stomach and promotes its cellular uptake via receptor-mediated endocytosis.	This variant is suggested to increase TCN1 expression, which is associated with lower vitamin B12 levels. This is due to TCN1 carrying 80–90% of serum B12, with the rest being carried by TCN2 to the brain and other organs. Upregulation of TCN1 lowers the B12 available for TCN2 [59].
GCLC ^1,2,3^; 446 + 233G>A	GCLC is the first rate-limiting step to produce glutathione. This enzyme catalyzes the reaction that combines glutamate and cysteine to produce gamma-glutamyl-cysteine.	While the full scope of this variant’s effects is not currently elucidated, it is associated with decreased glutathione synthesis. Therefore, it is likely a mechanism in which the enzymatic activity of GCLC is affected [60].
GSTP1 ^5^; 105I>V	GSTP1 encodes one of the glutathione transferase enzymes. This enzyme is involved in the attachment of toxins, carcinogens, and drugs to a reduced glutathione molecule.	This variant is associated with lower enzymatic activity, specificity for substrate, and thermal stability. This decreases the ability to conjugate reduced glutathione with toxins for removal [61].
HLA-DQ2.5 ^6^; 567C>T	HLA plays an important role in antigen presentation to CD4+ T cells. HLA-DQ2.5 presents gluten peptides to these immune cells.	The HLA-DQ2.5 variant has a conformation that leads to an increased immunological response to the presentation of gluten peptides. This is associated with an increased risk of developing celiac disease [62].
PEMT ^1,2,3,4,5^; 175V>M	PEMT is necessary for the conversion of phosphatidylethanolamine to phosphatidylcholine, which is the precursor to choline. Choline is needed to make acetylcholine and phospholipid membranes through its activity as a methyl donor.	This variant is associated with decreased enzymatic activity, lower phosphatidylcholine synthesis and, therefore, lower choline levels. Alterations in choline levels have been demonstrated in individuals with ASD, as well as downstream effects on methylation pathways, due to PEMT’s role as a methyl donor [63,64].

## Data Availability

Relevant genomics and data are presented in the paper. Full access to the genomic results is part of an online resource available to ordering clinicians and is not available in downloadable or printable form for patient privacy.
